# Free Radical Scavenging and Cellular Antioxidant Properties of Astaxanthin

**DOI:** 10.3390/ijms17010103

**Published:** 2016-01-14

**Authors:** Janina Dose, Seiichi Matsugo, Haruka Yokokawa, Yutaro Koshida, Shigetoshi Okazaki, Ulrike Seidel, Manfred Eggersdorfer, Gerald Rimbach, Tuba Esatbeyoglu

**Affiliations:** 1 Institute of Human Nutrition and Food Science, University of Kiel, Hermann-Rodewald-Straße 6, D-24118 Kiel, Germany; dose@foodsci.uni-kiel.de (J.D.); seidel@foodsci.uni-kiel.de (U.S.); rimbach@foodsci.uni-kiel.de (G.R.); 2 School of Natural System, Kanazawa University, Kakuma-machi, Kanazawa 920-1192, Japan; s-matsugoh@se.kanazawa-u.ac.jp (S.M.); haruside8133@gmail.com (H.Y.); koshi04015087@gmail.com (Y.K.); 3 Medical Photonics Research Center, Hamamatsu University School of Medicine, Handamachi 1-20-1, Higashi-ku, Hamamatsu, Shizuoka 431-3192, Japan; okazaki@hama-med.ac.jp; 4 DSM Nutritional Products, P.O. Box 2676, 4002 Basel, Switzerland; manfred.eggersdorfer@dsm.com

**Keywords:** astaxanthin, free radical scavenging, antioxidant, electron spin resonance spectroscopy

## Abstract

Astaxanthin is a coloring agent which is used as a feed additive in aquaculture nutrition. Recently, potential health benefits of astaxanthin have been discussed which may be partly related to its free radical scavenging and antioxidant properties. Our electron spin resonance (ESR) and spin trapping data suggest that synthetic astaxanthin is a potent free radical scavenger in terms of diphenylpicryl-hydrazyl (DPPH) and galvinoxyl free radicals. Furthermore, astaxanthin dose-dependently quenched singlet oxygen as determined by photon counting. In addition to free radical scavenging and singlet oxygen quenching properties, astaxanthin induced the antioxidant enzyme paroxoanase-1, enhanced glutathione concentrations and prevented lipid peroxidation in cultured hepatocytes. Present results suggest that, beyond its coloring properties, synthetic astaxanthin exhibits free radical scavenging, singlet oxygen quenching, and antioxidant activities which could probably positively affect animal and human health.

## 1. Introduction

**Figure 1:**
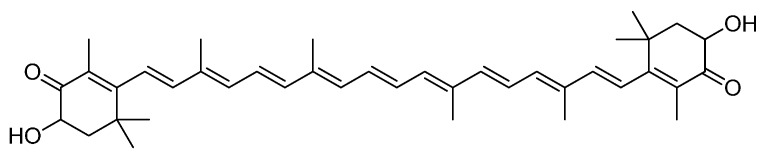
Chemical structure of astaxanthin.

Astaxanthin (3,3′-dihydroxy-β,β′-carotene-4,4′-dione, for chemical structure see Figure 1) is a xanthophyll carotenoid [1] which naturally occurs in algae, krill, trout, crayfish, and salmon. Astaxanthin is widely used in aquaculture nutrition as a coloring agent [2]. In addition to its coloring properties, astaxanthin may also affect immune status and reproduction [3,4].

Astaxanthin has two chiral centers at positions 3 and 3'. The astaxanthin stereoisomer -*3S*,*3S′*- is the main form found in wild salmon [5]. Most astaxanthin used in aquaculture nutrition is produced synthetically which yields three different stereoisomers, including *3S*, *3′S*; *3R*, *3′S*; and *3R*, *3′R* [1]. In 1981, Widmer *et al.* [6] synthesized astaxanthin from the educt 6-*oxo*-isophorone (3,5,5-trimethyl-2-cyclohexene-1,4-dione) via a seven-step synthesis. Over a Wittig reaction of two equivalents C15-phosphonium salt with C10-dialdehyde, astaxanthin was obtained with yields up to 50% yields [6].

Astaxanthin has also significant applications in the nutraceutical industry [7]. Various health benefits, including cardiovascular disease and cataract prevention, have been associated with astaxanthin consumption [3,8,9,10].

Although it has been suggested that potential health benefits of astaxanthin are, at least partly, mediated by its free radical scavenging [11], antioxidant [12,13], and gene-regulatory properties [14,15], systematic studies are scarce.

In previous studies, free radical scavenging activity of astaxanthin has been determined by FRAP (ferric reducing antioxidant power) [16], TEAC (trolox equivalent antioxidant capacity) [17], and ORAC (oxygen radical absorbance capacity) [18] assays, respectively. Furthermore, the decolorization of the 2,2-diphenyl-1-picrylhydrazyl (DPPH) radical may be used to assess the free radical scavenging activity of antioxidants, taking into account that alcohols as solvents may lead to an overestimation of its free radical scavenging activity [19,20,21]. The different antioxidant test systems, as described in the literature, differ in terms of underlying methodology, reagents, wavelength, pH, *etc.* It is suggested that a combination of various assays should be used in assessing antioxidant activities *in vitro* [22].

In addition to these rather indirect methods, electron spin resonance spectroscopy (ESR) combined with spin trapping has been established as a robust method for the direct assessment of free radical reactions [23]. Therefore, in this study, we applied ESR, as well as photon counting methods, to determine the free radical scavenging and singlet oxygen quenching activity of astaxanthin.

Furthermore, we studied the cellular antioxidant activity of astaxanthin in cultured hepatocytes. We addressed the question, if and to what extend astaxanthin may induce enzymatic antioxidant defense mechanisms, including paraoxoanase-1. Furthermore, we determined cellular glutathione levels in response to an astaxanthin treatment, since glutathione is the most important endogenous cytosolic antioxidant centrally involved in redox signaling and stress response.

Overall, this manuscript aims at providing comprehensive information in terms of the free radical scavenging, antioxidant, and cell signaling modifying properties of astaxanthin.

## 2. Results and Discussion

**Figure 2:**
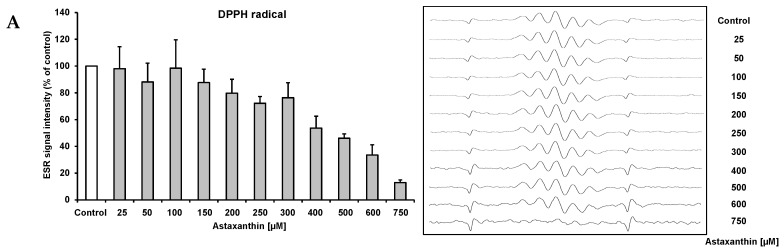

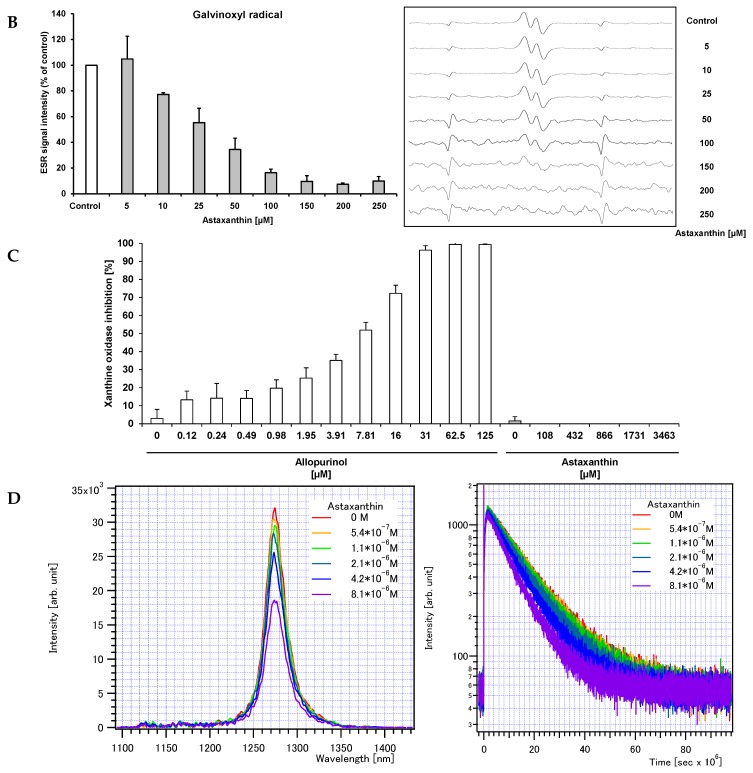
Scavenging effects of astaxanthin on DPPH radical (**A**), galvinoxyl radical (**B**), xanthine oxidase inhibition (**C**), and quenching of singlet oxygen (**D**). (**A**) The reaction mixture contained 500 μM DPPH and the given concentration of astaxanthin. All values are means + SD (experiments performed in triplicate); (**B**) Various astaxanthin concentrations were mixed with 500 µM galvinoxyl. Changes in the radical signal intensity are shown on the right side of the figure. All values are means + SD (three independent experiments performed in triplicate); (**C**) The reaction mixture contained 5 U/mL xanthine oxidase in 50 mM potassium phosphate buffer and the given astaxanthin concentrations. Allopurinol was used as a positive control. All values are means + SD (three independent experiments performed in triplicate); (**D**) Singlet oxygen quenching activity of astaxanthin as a function of concentration (5.4 × 10^−7^, 1.1 × 10^−6^, 2.1 × 10^−6^, 4.2 × 10^−6^, and 8.1 × 10^−6^ M astaxanthin), wavelength (**left**), and time (**right**). Photo emission was determined by a photon complex after laser irradiation at 532 nm in the presence of the test sample. Two independent experiments performed in duplicate.

We applied ESR and spin trapping in order to directly quantify the free radical scavenging activity of astaxanthin. Astaxanthin dose-dependently scavenged DPPH (Figure 2A) and galvinoxyl (Figure 2B) free radicals. However, astaxanthin did not scavenge superoxide anion free radicals (data not shown). Accordingly, astaxanthin did not inhibit xanthine oxidase, which generates superoxide anion free radicals (Figure 2C). When singlet oxygen is relaxed to the ground state, oxygen photon emission is observed. Singlet oxygen quenching activity of astaxanthin as a function of emission spectrum, concentration, and decay curve is given in Figure 2D. As shown in Figure 2D, astaxanthin dose-dependently quenched singlet oxygen as determined by photon counting.

**Figure 3:**
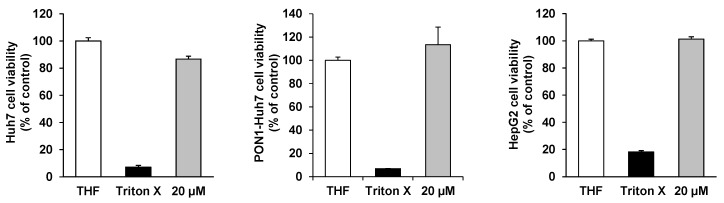
Effects of astaxanthin on cell viability in Huh7, PON1-Huh7, and HepG2 cells after 24 h incubation. Data are means + SD of at least two experiments performed in triplicate.

In our cell culture studies, astaxanthin did not exhibit any significant cytotoxicity in concentrations of up to 20 µM in Huh7, PON1-Huh7, and HepG2 as summarized in Figure 3. TritonX was used as a control to induce cytotoxicity as determined by the neutral red assay.

**Figure 4:**
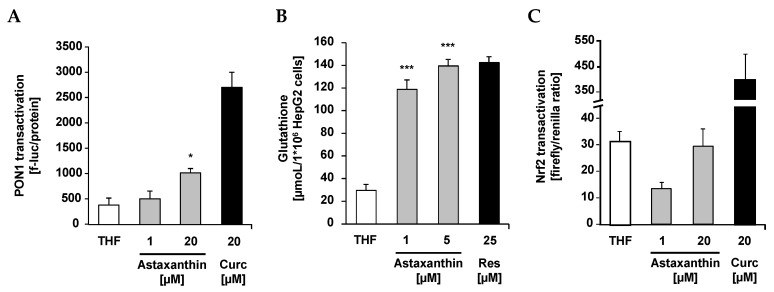
Effects of astaxanthin on transactivation of paraoxonase-1 (PON1) (**A**), cellular glutathione (GSH) levels (**B**) and transactivation of Nrf2 (**C**) in cultured hepatocytes. (**A**) PON1-Huh7 cells were seeded at a density of 0.15 × 10^6^ cells/well into 24 well plates and incubated for 24 h at 37 °C. Cells were treated with 1 and 20 µM astaxanthin. PON1 transactivation was measured after 48 h incubation of the cells with synthetic astaxanthin. Curcumin (Curc; 20 µM) was used as a positive control; (**B**) HepG2 cells were seeded at a density of 0.15 × 10^6^ cells/well into 24 well plates and incubated for 24 h. Cells were treated with 1 and 5 µM astaxanthin and incubated for an additional 24 h. Resveratrol (Res; 25 µM) was used as a positive control; (**C**) Huh7 cells were seeded at a density of 0.15 × 10^6^ cells/well into 24 well plates and incubated for 24 h at 37 °C. Cells were transfected for 24 h. Nrf2 transactivation was measured after 24 h incubation of the cells with 1 and 20 µM astaxanthin. Curcumin (Curc; 20 µM) was used as a positive control. All values are means + SEM (two independent experiments performed in triplicate), statistical significant differences between THF-control cells and astaxanthin supplemented cells are indicated as * *p* < 0.05, *** *p* < 0.001; one-way ANOVA LSD (Figure 4A) and Dunnett-T (Figure 4B).

Supplementation of cultured PON1-Huh7 cells with 20 µM synthetic astaxanthin resulted in a moderate induction of paraoxoanse-1 (PON1) (Figure 4A). Furthermore, synthetic astaxanthin dose-dependently increased cellular glutathione (GSH) levels in HepG2 cells (Figure 4B). The increase in cellular GSH was not accompanied by an increase in Nrf2 transactivation as shown in Figure 4C. Curcumin and resveratrol were used as positive controls, respectively.

Additionally, mRNA levels of *Gclc* (glutamate-cysteine ligase, catalytic subunit) and other Nrf2-target genes, including *Gsta (*glutathione *S*-transferase alpha 1), *Gpx* (glutathione peroxidase), *Hmxo1* (heme oxygenase 1), *Nqo1* (NAD(P)H dehydrogenase [quinone] 1), *Sod1* (superoxide dismutase), and *Sod2*, were not significantly changed due to the astaxanthin treatment (Table 1).

**Table 1:** Nrf2-target gene expression in astaxanthin treated Huh7 cells (THF control cells were set to be 1.0).

Gene	Astaxanthin [µM]	
	1	20
*Gclc*	0.91 ± 0.08	1.03 ± 0.26
*Gsta*	1.01 ± 0.15	0.99 ± 0.18
*Gpx1*	1.34 ± 0.24	1.12 ± 0.30
*Hmxo1*	0.40 ± 0.04	0.85 ± 0.32
*Nqo1*	1.21 ± 0.18	1.32 ± 0.21
*Sod1*	1.22 ± 0.21	0.99 ± 0.18
*Sod2*	1.35 ± 0.18	1.39 ± 0.54

**Figure 5:**
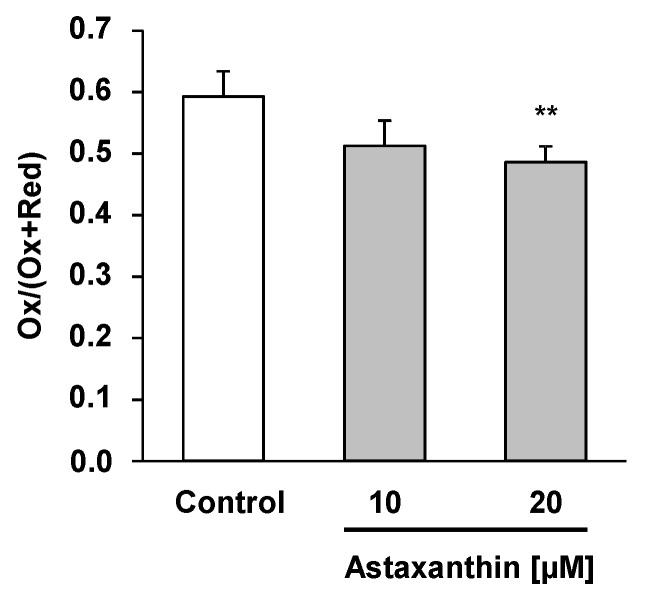
Impact of astaxanthin on the ability of human HepG2 cells to prevent cumene hydroperoxide/hemin stimulated lipid peroxidation. HepG2 cells were seeded at a density of 0.1 × 10^6^ cells/well into a 96-well plate and incubated for 24 h. Cells were treated with two concentrations of astaxanthin (10 and 20 µM) for an additional 24 h. Cells were rinsed and loaded with C11-BODIPY for 30 min and then exposed to cumene hydroperoxide (8 mM)/hemin (8 µM) for 1 h. Control cells were treated with cell culture medium only. Simultaneously, basal levels of lipid peroxidation were assessed for each treatment concentration (data not shown) and control. Lipid peroxidation was determined by examining the ratio of green (oxidized) and the sum of green and red (oxidized + non-oxidized) emissions using a fluorometric plate reader. Data are means + SD of at least two independent experiments performed in duplicate. ** *p* < 0.01, one-way ANOVA Games–Howell.

HepG2 cells were stressed with cumene hydroperoxide/hemin to provoke lipid peroxidation. We applied the BODIPY assay in order to determine lipid peroxidation in our HepG2 hepatocytes following the astaxanthin treatment. The BODIPY probe exhibits a change in fluorescence after interaction with peroxyl groups. Under the conditions investigated, supplementation of HepG2 cells with 10 and 20 µM astaxanthin significantly decreased lipid peroxidation in HepG2 cells (Figure 5).

In the present study, we observed a dose-dependent free radical scavenging activity of astaxanthin in terms of DPPH and galvinoxyl free radicals as determined by ESR and spin trapping. Current ESR data regarding the free radical scavenging activity of astaxanthin in terms of DPPH free radicals are in line with literature data [24,25], where different photometric assay systems were applied, e.g., DPPH, ferric reducing antioxidant power and hydroxyl radical scavenging.

The free radical scavenging activity of astaxanthin is suggested to be mediated by electron transfer, radical adduct formation and hydrogen atom transfer [26]. Singlet oxygen quenching of carotenoids, including astaxanthin is mediated by energy transfer between the electrophilic singlet oxygen and the polyene backbone. In general, the singlet oxygen quenching activity of carotenoids increases with increasing length of conjugation [27]. Furthermore, our BODIPY data suggest that astaxanthin significantly prevents lipid peroxidation. The prevention of lipid peroxidation due to astaxanthin may improve the sensory quality and shelve life of fish which warrants further investigation. However, it needs to be taken into account that the astaxanthin concentrations, as administered in our cell culture assays, may be higher than astaxanthin concentrations in fish plasma [28].

We did not observe any inhibition of xanthine oxidase in response to the astaxanthin treatment. Thus, unlike other antioxidants including flavonoids [29,30], astaxanthin does not seem to mediate antioxidant activity via xanthine oxidase inhibition.

For our cell studies, astaxanthin was dissolved in tetrahydrofuran (THF). We used THF since it has been previously shown by our group to be non-cytotoxic to HepG2 cells [31]. Furthermore, astaxanthin dissolved in THF exhibits an appropriate stability in the cell culture medium [31] and is effectively taken up by HepG2 and HT29 cultured cells [31,32].

Current cell culture data clearly suggest that synthetic astaxanthin does not only work as a free radical scavenger, but also as an inductor of cellular antioxidant defense mechanisms, including PON1 and reduced glutathione. PON1 is a hepatic enzyme which prevents and/or delays the oxidation of LDL [33]. Thus, the prevention of plasma lipid peroxidation in humans due to astaxanthin, as reported by Barlic and co-workers [34], may be partly mediated by an induction of paraoxonase-1. Recently, it has been shown that PON1 also counteracts oxidative stress induced by mercury chloride, thereby offering a beneficial strategy against HgCl_2_ toxicity [35] which may be of particular interest for fish nutrition. We also determined the cellular activity of natural astaxanthin which was isolated from *Haematococcus pluvialis* (data not shown). Importantly, natural source astaxanthin did not exhibit a superior bioactivity in any of our test systems as also stated by others [36]. In terms of PON1, a transactivation occurred only in response to synthetic astaxanthin.

Synthetic astaxanthin dose-dependently increased cellular GSH levels in HepG2 cells which is in line with findings by Saw and co-workers who found an induction of cellular GSH due to both astaxanthin and omega-3 fatty acids [13]. Interestingly, in our study the increase in cellular GSH in HepG2 cells was, however, not accompanied by an increase in Nrf2 transactivation. Furthermore, *Gclc*, the rate limiting enzyme of GSH synthesis, was not induced by astaxanthin. Additionally, steady state levels of other Nrf2 target genes, including *Gsta*, *Gpx*, *Hmxo*, *Nqo1*, *Sod1*, and *Sod2* were not induced in response to the astaxanthin treatment. Thus, it is suggested that the increase in cellular GSH due to astaxanthin was not mediated by an Nrf2 dependent signal transduction pathway. It is worth mentioning that GSH synthesis is not only regulated by Nrf2, but also by other transcription factors, including activator protein 1 (AP-1) [37,38], specificity protein 1 (Sp1) [39,40], nuclear respiratory factor 1 (Nrf1) [41,42], and nuclear factor kappa-light-chain-enhancer of activated B cells (NFκB) [42,43]. Based on the present data, it is suggested that elevated GSH concentration may be a sign of lower oxidative stress levels as a result of the astaxanthin treatment.

## 3. Experimental Section

### 3.1. Radical Scavenging Measured by Electron Spin Resonance Spectroscopy (ESR)

ESR measurements were performed using a JEOL JES-FR30EX free radical monitor (JEOL Ltd., Akishima, Japan). The measurement conditions were as follows: magnetic field, 337.394 ± 7.5 mT; power, 4 mW; sweep time, 1 min; sweep width, 7.5; modulation, 100 kHz, 0.32 mT; amplitude, 400; and time constant, 0.3 s. Signal intensity was compared based on the ratio against the magnetic Mn^2+^ marker and was represented by relative height ratio. The ESR spectra were measured three times.

*Preparation of the astaxanthin stock solution.* Astaxanthin (DSM, Kaiseraugst, Switzerland) was dissolved in 21.8% tetrahydrofuran (THF) (Wako Chemicals, Osaka, Japan) and 78.2% ethanol (Nacalai Tesque, Kyoto, Japan). The actual concentration of astaxanthin was determined by diluting the stock solution with THF using the molar coefficient of astaxanthin (69,600 M^−1^·cm^−1^ at 477 nm) [44]. Based on this value, the stock solution of astaxanthin was determined as 30.56 mM. The astaxanthin stock solution was further diluted with *tert*-butyl alcohol/THF (89.3:10.7, *v*/*v*).

*DPPH radical scavenging experiments.* To a reaction mixture containing 80 µL distilled water, 20 µL 500 µM DPPH (in methanol; Nacalai Tesque, Kyoto, Japan) and 100 µL astaxanthin (0, 50, 100, 200, 300, 400, 500, 600, 800, 1000, 1200, and 1500 µM) were added and stirred for a few seconds. *tert*-Butyl alcohol (containing 10.7% THF) was used as a control. After 1 min incubation the ESR spectra were measured.

*Galvinoxyl radical scavenging experiments.* To a reaction mixture containing 80 µL distilled water, 20 µL 500 µM galvinoxyl (in methanol; Nacalai Tesque, Kyoto, Japan) and 100 µL astaxanthin (0, 10, 20, 50, 100, 200, 300, 400, 500 µM) were added and stirred for a few seconds. *tert*-Butyl alcohol (containing 10.7% THF) was used as a control. After 1 min incubation the ESR spectra were measured.

*Superoxide radical scavenging experiments.* To the reaction mixture of 30 µL of 5 mM hypoxanthine (dissolved in NaOH and then diluted with PBS (pH 7.4)), 40 µL 4 M 5,5-dimethyl-1-pyrroline *N*-oxide (DMPO), 25 µL ultrapure water, and 10 µL of astaxanthin (0, 1, 7.5, 15 mM) were added to 5 µL of 1 U/mL xanthine oxidase in 200 mM PBS buffer solution (pH 7.4). After 1 min incubation at 25 °C the ESR spectra were measured. Three independent experiments were performed in triplicate.

### 3.2. Inhibition of Xanthine Oxidase

The inhibition of xanthine oxidase was measured according to Bräunlich *et al.* 2013 [45] with some modifications. An enzyme solution consisting of 400 µL xanthine oxidase (5 U/mL) in 7.6 mL 50 mM potassium phosphate buffer (pH = 7.5) was prepared immediately before use. One hundred and fifty microliters of the prepared enzyme solution was added to each well of a 96 well microtiterplate. Astaxanthin (4 g/L in THF) was serially diluted in the wells (dilution factor 2). THF was used as blank sample. Allopurinol (500 µM; Sigma, Darmstadt, Germany) served as positive control and was serially diluted in the wells (dilution factor 2). The substrate hypoxanthine (40 µg/mL; dissolved in NaOH and then in distilled water) was prepared daily and 80 µL were injected at cycle 10. The absorption was measured at 290 nm (microplate reader Tecan infinite 200Pro, Crailsheim, Germany) over 110 cycles (≈150 s) at 28 °C. At least three independent experiments were performed in triplicate.

### 3.3. Quenching Experiments of Near-Infrared Emission Spectra of ^1^O_2_ by Astaxanthin

The singlet oxygen scavenging activity of astaxanthin was determined by photon counting according to Shimizu *et al.* (2010) [46] with some modifications.

Ten millimolar astaxanthin was prepared in THF/ethanol (20/80; *v*/*v*). After preparing 10 mM astaxanthin, 1 mM astaxanthin was prepared in THF/ethanol (10.7/89.3; *v*/*v*). The final concentration of astaxanthin was confirmed by measuring the solution of 2 mL ethanol and 80 μL astaxanthin solution (1 mM) by UV–Vis spectrometer (Hitachi, Tokyo, Japan) using the molar coefficient of astaxanthin (69,600 M^−1^·cm^−1^ at λ = 477 nm).

The ^1^O_2_ formation was directly measured by the near-infrared luminescence at around 1270 nm from deactivated ^1^O_2_ which corresponds to the ^1^O_2_ (^1^Δ_g_)–^3^O_2_ (^3^Σ_g_^−^) transition. The emission from ^1^O_2_ was measured using an apparatus based on a commercially available apparatus and improved for high-sensitivity detection (NIR-PII System, Hamamatsu Photonics K.K., Hamamatsu, Japan). The excitation pulse was obtained using a dye laser (CL-EGC USHO Optical Systems Co., Ltd., Osaka, Japan) excited by an Nd-YAG Laser (NL 240/TH EKSPLA Lithuania). The excitation wavelength was 530 nm, pulse width and intensity were approximately 7 ns and 17 mW, respectively, and the repetition rate was 500 Hz. Emission of ^1^O_2_ was monitored using an infrared-gated image intensifier (NIR-PII, Hamamatsu Photonics) after passage through a polychromator (250 is, Chromex Inc., Albuquerque, NM, USA). Measurements started at 1 μs after application of the excitation pulse and the exposure time was 50 μs. Signals were accumulated by repeated detection (500 Hz, 10 s). The emission decay was detected by the NIR-PII system (Hamamatsu Photonics) after passing through the bandpass filter (1270 nm) and recorded by the multichannel scalar (NanoHarp 250, PicoQuant GmbH, Berlin, Germany). The measuring time was set at 16 ns/bin for ca. 260 μs and accumulated for 2 min at 500 Hz. The solution studies consist of 2 mL of 5 μM Rose Bengal (in ethanol) as a photosensitizer. The quenching of the singlet oxygen decay was examined by adding 109 μM astaxanthin as follows: addition of 10 μL, 10 μL (final 20 μL), 20 μL (final 40 μL), 40 μL (final 80 μL), and 80 μL (final 160 μL). The decay curve and reaction rate of astaxanthin were calculated using the decay curve and quenching ratio of the duplicate experiments.

### 3.4. Cell Culture

Human Huh7 hepatic cellular carcinoma cells (Institute of Applied Cell Culture, Munich, Germany) were cultivated in high glucose (4.5 g/L) Dulbecco’s modified Eagle’s medium (DMEM, with sodium pyruvate, l-glutamine and 3.7 g/L NaHCO_3_ supplemented with 10% (*v*/*v*) fetal bovine serum (FBS), 100 U/mL penicillin and 100 µg/mL streptomycin (all PAN-Biotech, Aidenbach, Germany) at 37 °C in a 5% CO_2_ setting. For the preparation of PON1-Huh7 cells, Huh7 cells were stably transfected with a 1009 bp [−1013, −4] fragment of the human PON1 promoter (kindly provided by X. Coumoul and R. Barouki, INSERM UMR-S, Paris, France [47]). PON1-Huh7 cells were cultured in high glucose (4.5 g/L) DMEM (without l-glutamine) supplemented with 10% heat-inactivated FBS, 2 mmol/L glutamine, 100 U/mL penicillin, 100 µg/mL streptomycin, and 100 µg/mL G418 sulfate (PAN-Biotech, Aidenbach, Germany) in a humidified atmosphere of 5% CO_2_ at 37 °C. Human HepG2 hepatocellular carcinoma cells (Institute of Applied Cell Culture, Munich, Germany) were cultured in RPMI 1640 (with l-glutamine and 2.0 g/L NaHCO_3_; PAN-Biotech, Aidenbach, Germany) containing 10% FBS, 100 U/mL penicillin and 100 µg/mL streptomycin in an incubator (95% air, 5% CO_2_) at 37 °C. At least two independent experiments were performed in triplicate. All cell culture experiments were conducted at non-cytotoxic concentrations of astaxanthin.

### 3.5. Neutral Red Cell Viability Assay

PON1-Huh7, Huh7, and HepG2 cells were seeded at a density of 1.5 × 10^5^ cells per well in 24-well plates. Cells were treated with 1–20 µM astaxanthin for 48 (PON1-Huh7) or 24 h (Huh7, HepG2). Afterwards, the culture medium was replaced with neutral red solution (50 μg/mL, prepared in cell culture medium) and incubated for 2 h at 37 °C. Cells were extracted with ethanol:water:glacial acetic acid (50:49:1, *v*/*v*/*v*; glacial acetic acid purchased from Carl Roth, Karlsruhe, Germany) and incubated at room temperature for 15 min under continuous shaking. The absorbance was measured at 540 nm in a plate reader (Labsystems iEMS Reader, Helsinki, Finland). At least two independent experiments were performed in triplicate.

### 3.6. Paraoxonase Activity

PON1-Huh7 cells were seeded at a density of 0.15 × 10^6^ cells per well in 24-well plates and incubated for 24 h. Subsequently, cells were treated with 1 and 20 µM synthetic (DSM, Kaiseraugst, Switzerland) *versus* algae-based (BioAstin, Cyanotech Corporation, Kailua-Kona, HI, USA) astaxanthin. Curcumin (20 µM) served as positive control. After 48 h incubation, cells were washed with PBS and lysed with cell culture lysis reagent (1:5, *v*/*v*; Promega, Mannheim, Germany). Luciferase activity (Luciferase Assay System; Promega, Mannheim, Germany) was measured by luminescence reading (Infinite 200 microplate reader, Tecan, Crailsheim, Germany). Results were normalized to total protein content, determined by the BCA assay (Pierce, Bonn, Germany) according to the manufacturer’s instructions. At least two independent experiments were performed in triplicate.

### 3.7. Glutathione Assay

HepG2 cells (0.15 × 10^6^ cells/well) were incubated for 24 h at 37 °C. Cells were treated with 1 and 5 µM astaxanthin, respectively and incubated for an additional 24 h. Glutathione measurements were performed according to Esatbeyoglu *et al.* (2014) [48] and Vandeputte *et al.* (1994) [49].

### 3.8. Nrf2 Transactivation

Huh7 cells (0.15 × 10^6^ cells per well) were incubated in 24-well plates for 24 h. Cells were transiently transfected with pARE_GIGPx_Luc according to Wagner *et al.* (2010) [50]. After 24 h-transfection, cells were incubated with 1 and 20 µM astaxanthin for further 24 h. Curcumin (20 µM) served as positive control. Cell lysis and measurement of Nrf2 transactivation were conducted as reported previously [50]. At least two independent experiments were performed in triplicate.

### 3.9. RNA Isolation and qRT-PCR

Cells were harvested with TriFAST (VWR International GmbH, Erlangen, Germany) and RNA was isolated according to the manufacturer’s protocol. RNA concentration and quality were determined using the NanoDrop ND-2000 spectrophotometer (VWR International GmbH, Erlangen, Germany). Aliquots of RNA samples were stored at −80 °C until PCR analyses. mRNA expression levels of glutathione peroxidase 1 (*Gpx1*), superoxide dismutase 1, soluble (*Sod1*), superoxide dismutase 2, mitochondrial (*Sod2*), heme oxygenase 1 (*Ho-1*), NAD(P)H dehydrogenase [quinone] 1, (*Nqo1*), glutamate-cysteine ligase, catalytic subunit (*Gclc*), glutathione *S*-transferase alpha 1 (*Gsta*), and glyceraldehyde-3-phosphate dehydrogenase (*Gapdh*) were determined using target-specific primers. Primer3 Input software version 0.4.0 was used for primer design (http://bioinfo.ut.ee/primer3-0.4.0/primer3/). Primer sequences are depicted in Table 2. Primers were purchased from Eurofins MWG (Ebersberg, Germany). qRT-PCR was carried out as a one-step procedure using the SensiFAST™ SYBR No-ROX One-Step Kit (Bioline, Luckenwalde, Germany) with SYBR Green detection on a Rotorgene 6000 cycler (Corbett Life Science, Sydney, Australia). Calculation of results was done by an external standard curve. Transcription levels of target genes were normalized by the transcription of *Gapdh* which served as housekeeping gene in qRT-PCR analyses.

### 3.10. Lipid Peroxidation Determined by BODIPY Assay

C11-BODIPY (581/591) (Life technologies, Darmstadt, Germany) fluorescent dye served as a lipid peroxidation sensor. C11-BODIPY is a lipophilic fatty acid analog which incorporates into biomembranes and shifts its fluorescent properties from reduced (λ_ex_ = 540 nm/λ_em_ = 595 nm) to oxidized (λ_ex_ = 480 nm/λ_em_ = 520 nm) status. Adjusted index of BODIPY oxidation was calculated as follows:

Adjusted Index = Em_Ox_/(Em_Ox_ + Em_Red_) (1)

HepG2 cells (density of 0.15 × 10^6^ cells per well in 24-well plates and incubated for 24 h) were incubated with 10 and 20 µM astaxanthin for 24 h. Cells were treated with 10 µM C11-BODIPY (581/591) in medium without FBS for 30 min. BODIPY was removed and cells were stressed with 80 µM cumene hydroperoxide/80 nM hemin in PBS for 1 h. Afterwards, PBS was refreshed and fluorescence was measured in adherent cells.

### 3.11. Statistical Analysis

Statistical analysis was conducted using SPSS software version 23.0 (SPSS Inc., Munich, Germany). All cell culture data were tested for normal distribution (Kolmogorow–Smirnow). One-way ANOVA was applied. In case of homogeneous variances, LSD and Dunnett-T *post hoc* test and, in case of heterogeneous variances, Games–Howell *post hoc* test was conducted to test significant differences between the control group and the test groups. All data are expressed as the mean + SD or SEM. Significance was accepted at *p* < 0.05.

**Table 2:** Nucleotide sequences and annealing temperatures of primers used in qRT-PCR analyses in cultured Huh7 cells.

Gene	Gene-ID	Description	Primer, Forward (5′–3′)	Primer, Reverse (5′–3′)	Annealing (°C)
*Gpx1*	2876	glutathione peroxidase 1	ACACCCAGATGAACGAGCTG	CCGGACGTACTTGAGGGAAT	58
*Sod1*	6647	superoxide dismutase 1, soluble	GGGGAAGCATTAAAGGACTG	CAACATGCCTCTCTTCATCC	55
*Sod2*	6648	superoxide dismutase 2, mitochondrial	GCACTAGCAGCATGTTGAGC	GATCTGCGCGTTGATGTG	55
*Hmox1*	3162	heme oxygenase 1	CCA GGC AGA GAA TGC TGA GT	GTA GAC AGG GGC GAA GAC TG	59
*Nqo1*	1728	NAD(P)H dehydrogenase [quinone] 1	CTG ATC GTA CTG GCT CAC TC	GAA CAG ACT CGG CAG GAT AC	58
*Gclc*	2729	glutamate-cysteine ligase, catalytic subunit	TTT GGT CAG GGA GTT TCC AG	TGA ACA GGC CAT GTC AAC TG	59
*Gst*	2938	glutathione *S*-transferase alpha 1	CGTATGTCCACCTGAGGAAA	GCCAACAAGGTAGTCTTGTCC	60
*Gapdh*	2597	glyceraldehyde-3-phosphate dehydrogenase	CAATGACCCCTTCATTGACC	GATCTCGCTCCTGGAAGATG	58

## 4. Conclusions

Collectively, our data suggest that, beyond its coloring properties, synthetic astaxanthin exhibits important free radical scavenging, oxygen quenching, and antioxidant activities. The direct free radical scavenging activity of astaxanthin was determined by ESR and spin trapping and its oxygen quenching activity by photon counting. The cellular antioxidant activity of astaxanthin was examined in various bioassays (e.g., transactivation of PON1, cellular GSH levels, transactivation of Nrf2 and expression of its target genes, lipid peroxidation) in cultured cell lines. Since we worked with cell lines, our findings can not be generalized to primary cells and animal models, respectively, and should therefore be verified in appropriate *in vivo* models in the future. Additionally, it would be interesting to test whether there are synergistic interactions between astaxanthin and other lipid (e.g*.*, vitamin E) and water soluble antioxidants (e.g., ascorbic acid, flavonoids) in terms of their free radical scavenging, antioxidant and gene-regulatory activity.
